# A New and Environmentally Friendly Route for Preparation of Carbon Microspheres from Wheat Straw

**DOI:** 10.1155/2013/146930

**Published:** 2013-10-29

**Authors:** Chen Leishan, Wang Cunjing, Miao Yu, Chen Gairong

**Affiliations:** College of Chemistry and Chemical Engineering, Xinxiang University, Xinxiang 453003, China

## Abstract

The reactions were performed to synthesize carbon materials using wheat straw powder as raw material. The wheat straw powder was first hydrolyzed at the absence of a catalyst at 190°C for 1 h, then the hydrolyzate solution was used as carbon source to prepare carbon materials via hydrothermal carbonization at 180°C in the absence of a catalyst for 8 h. The influence of solid-liquid-ratio of wheat straw to water on the morphology of the product was investigated. The samples were examined by a scanning electron microscope and Fourier transform infrared spectroscopy. The results show that the product was carbon microspheres with a large number of O–H, CHO, and other functional groups, and the diameters of carbon microspheres noticeably depended on the solid-liquid ratio. When the solid-liquid ratio was 1 : 60, the diameters of carbon microspheres were in the range of 100 to 300 nm when the solid-liquid ratio was 1 : 40, carbon microspheres with larger and more uniform diameters mostly about 250 nm were obtained, and when the solid-liquid-ratio was 1 : 20, there were more larger carbon microspheres with diameters about 800 nm in the product and the surface of these carbon microspheres is smoother, whereas; the uniformity of the product deteriorates.

## 1. Introduction

In recent years, carbonaceous materials have attracted much interest across the world due to their special atomic configurations and important applications. As a member of the carbon family, carbon microspheres are becoming an interesting research object for many researchers due to their potential applications in many fields. Some methods have been developed for the synthesis of carbon microspheres such as emulsion method, suspension method, and chemical vapor deposition; however, in these methods the main carbon source fossil resources like pitch, acetylene, benzene and so on which can be refined from oil. Facing increasing crude oil prices and the unclear availability of fossil resources, on the long run, it is an attractive option to create carbonaceous materials from renewable resources. Biomass is becoming a qualification, as a promising starting material, for the synthesis of carbonaceous materials because of its low value, huge amount, rapid regeneration, easy access, and environmental friendship. For previous studies, many researchers have synthesis carbon microspheres via the hydrothermal carbonization process of pure glucose [1, 2], which can provide theoretical fact for the production carbonaceous materials using biomass. 

Progress has been made on the preparation of carbonaceous materials using biomass [3–8]; however, only a little research has been done to synthesize and recognize the structure of carbon microspheres based on natural resources. Wang et al. [9] prepared carbon microspheres using rice husk as carbon source and sulfuric acid as the suitable reagent for the high hydrolysis rate of rice husk via hydrothermal carbonization at low temperature, but sulfuric acid is not environmentally friendly. Therefore, we will focus on a more friendly approach to synthesize carbon microspheres using biomass as carbon source.

On the basis of the previous work, in this paper, the reactions were performed to synthesize carbon microspheres using wheat straw powder as raw material via hydrothermal carbonization at low temperature 180°C. The wheat straw powder was first hydrolyzed in the absence of a catalyst under the subcritical water at 190°C, and then the hydrolyzate solution was used as carbon source to prepare carbon microspheres via hydrothermal carbonization. The whole process proceeded in a sealed reaction container without catalyst additives, no pollutant generation. Besides, in retrospect the hydrothermal carbonization process, an environmentally friendly route that uses water as solvent, has the advantage to synthesize functional carbonaceous materials with abundant functional groups remaining on the product surface, which can further widen the application field of carbon microspheres.

## 2. Experimental

Wheat straw was washed with distilled water, then dried at 60°C in an oven and porphyrized to 50 mesh. Then an appropriate amount of wheat straw powder was mixed with distilled water, and the ratio of wheat straw powder mass (g) to distilled water was 1 : 60, 1 : 40, and 1 : 20, respectively.

The hydrolysis reaction under different solid-liquid ratio was kept in a 50 mL stainless steel reactor at 190°C for 1 h in an oven. Then the solid wheat straw residue and liquid were separated by centrifugal separation. The hydrolysis solution was loaded onto a 50 mL stainless steel reactor for synthesis of carbon microspheres. Then the hydrothermal reaction under different solid-liquid ratio was then kept at the same temperature 180°C for 8 h. After reaction, the solid product was collected, washed with ethanol and distilled water till neutral, and then dried at 80°C in an oven. The morphology and the size of the samples were examined using a scanning electron microscope (SEM); the functional groups were examined by FT-IR spectroscopy, the samples as powder-pressed KBr pellets.

## 3. Results and Discussion

**Figure 1:**
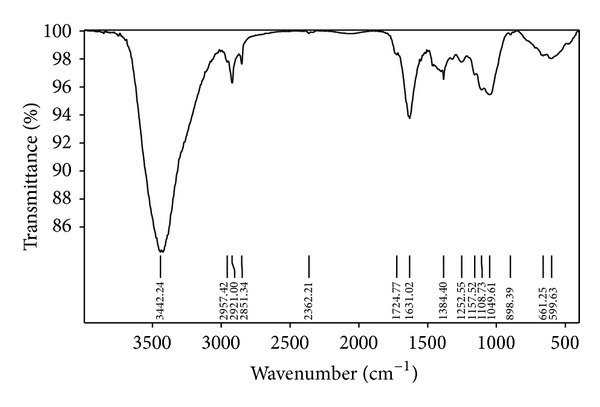
FT-IR spectrum of wheat straw.

Figure 1 shows the FT-IR spectrum of wheat straw, from which it can be observed that the bands in the spectrum mainly corresponded to stretching vibrations of O–H, C–H, C–O, and C=O bonds [10]. The band at approximately 3442.24 cm^−1^ corresponded to stretching vibrations of O–H bond. The bands in the range 2957.42–2853.17 cm^−1^ were assigned to C–H bond, whereas the bands in the range 1724.77–1631.02 cm^−1^ were attributed to C=O bond vibrations. The band at 1384.40 cm^−1^ probably corresponds to stretching vibrations of C–O or C–H. The bands in the range 1252.55–1157.52 cm^−1^ were attributed to benzene hydroxyl C–O bond of lignin or C–O bond of carbonhydrates, whereas, the bands in the range 1157.52–1047.27 cm^−1^ mainly correspond to vibrations of O–H and C–O–C, probably due to P–O–C and Si–O bond.

**Figure 2:**
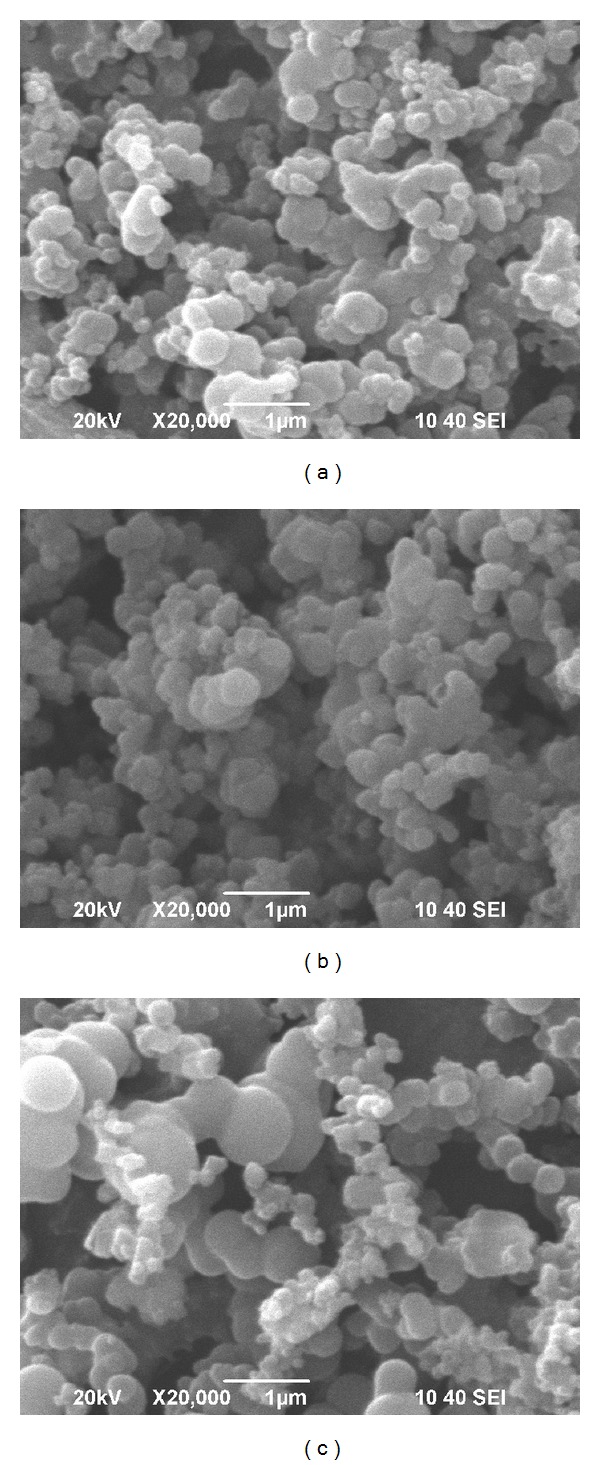
SEM images of the product (a) 1 : 60, (b) 1 : 40, and (c) 1 : 20.

Figure 2 shows the SEM images of the product under different solid-liquid ratio.  Figure 2(a) depicts the image of the product under solid-liquid ratio 1 : 60, from which it can be seen that the product was carbon microspheres with smooth surface and diameters in the range of 100 to 300 nm.  Figure 2(b) depicts the SEM image of the product under solid-liquid ratio 1 : 40; in contrast to Figure 2(a), the observation demonstrates that these carbon microspheres have larger and more uniform diameters mostly about 250 nm.  Figure 2(c) depicts the SEM image of the product under solid-liquid ratio 1 : 20, it can be obviously seen that there were more larger carbon microspheres with diameters about 800 nm in the product and the surface of these carbon microspheres is smoother, whereas the uniformity of the product deteriorates.

It was found that the diameters of carbon microspheres noticeably depended on the solid-liquid ratio. Under low solid-liquid ratio, corresponding to raw materials and hydrolyzate concentration, less precursors were carbonized, and carbon microspheres were formed with small diameters. With the increase of solid-liquid ratio, the corresponding raw materials and hydrolyzate concentration increased, and more precursors carbonized resulted in larger sized carbon microspheres formed; meanwhile, the uniformity of the product deteriorates due to no stirring in the progress.

**Figure 3:**
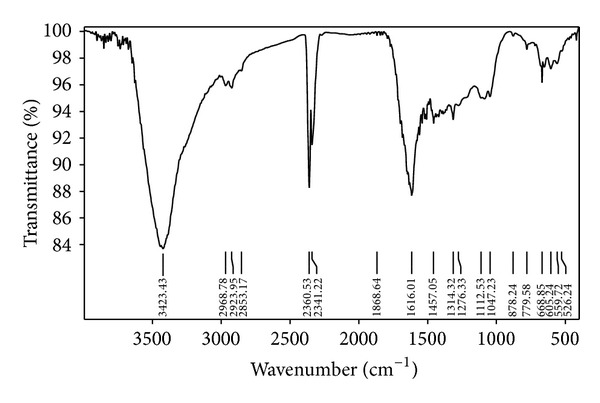
FT-IR spectrum of carbon microspheres.

Figure 3 shows the FT-IR spectrum of the product after hydrothermal reaction. In contrast to Figure 1, it can be observed that except that there was a strong peak at 3423.43 cm^−1^ which corresponds to stretching vibrations of O–H and other bands in the spectrum, a significant change is there with strong peak at 1616.01 cm^−1^ between 1457.05–1868.64 cm^−1^ attributed to C=C bond vibration of aromatic ring, indicating that hydrolysis solution had been carbonized. In addition, strong bands corresponding to stretching vibrations of CHO appeared in the spectrum implied that besides the existence of large numbers of residual groups of carbon resource in the product, there were other oxygen-containing groups in the product. The presence of these functional groups offered more possibility of further functionalization and made the materials applied in more fields.

## 4. Conclusion

The results demonstrate that carbon microspheres with a large number of O–H, CHO, and other functional groups can be prepared using wheat straw as carbon source via hydrothermal carbonization. The size and uniformity of the carbon microspheres was influenced by the solid-liquid ratio to a certain extent. Compared to previous work, in the synthesis route to prepare carbon microspheres using biomass as carbon source, no catalyst was used, greatly simplifying the subsequent separation. This work is of great significance to the theoretical studies and further industrialization using biomass to synthesize carbon materials.
